# Effect of Transcranial Direct Current Stimulation of the Medial Prefrontal Cortex on the Gratitude of Individuals with Heterogeneous Ability in an Experimental Labor Market

**DOI:** 10.3389/fnbeh.2017.00217

**Published:** 2017-11-03

**Authors:** Pengcheng Wang, Guangrong Wang, Xiaofei Niu, Huiliang Shang, Jianbiao Li

**Affiliations:** ^1^Business School, Tianjin University of Finance and Economics, Tianjin, China; ^2^Reinhard Selten Laboratory, Nankai University, Tianjin, China; ^3^Neural Decision Science Laboratory, Weifang University, Weifang, China; ^4^China Academy of Corporate Governance, Business School, Nankai University, Tianjin, China

**Keywords:** tDCS, mPFC, gratitude, social heterogeneity, gift exchange game

## Abstract

Gratitude is an important aspect of human sociality, which benefits mental health and interpersonal relationships. Thus, elucidating the neural mechanism of gratitude, which is only now beginning to be investigated, is important. To this end, this study specifies the medial prefrontal cortex (mPFC) involved in the gratitude of heterogeneous individuals using the transcranial direct current stimulation (tDCS) technique. Previous neural studies have shown the involvement of mPFC in social cognition and value evaluation, which are closely related to gratitude. However, the causal relationship between this neural area and gratitude has not been fully examined and the effect of individual social heterogeneity has been ignored. Meanwhile, behavioral economics studies have proposed that the abilities of employees in the labor market would affect their gratitude and emotional response. Thus, we designed an experiment based on gift exchange game to investigate the relationship between mPFC and gratitude of heterogeneous employees. Before the experiment, participants were asked to perform self-cognition of their abilities through an appropriately difficult task. We then used the effort of participants to imply their gratitude and analyzed the effort levels of employees with different abilities under anodal, cathodal, and sham stimulations. The results showed that employees under anodal stimulation were significantly likely to increase their effort than those under sham stimulation, and employees under cathodal stimulation ranked at the bottom of the list. Moreover, the effort levels of low-ability employees were obviously higher than those of high-ability employees. The cathodal stimulation of mPFC significantly reduced the effort levels of low-ability employees, whereas its anodal tDCS stimulation increased the effort levels of high-ability employees. These outcomes verify the relationship between mPFC and gratitude using tDCS and provided one of the first instances of neural evidence for the incentive mechanism design in the labor market to a certain extent.

## Introduction

Gratitude, an important part of societal orientation, is a person’s positive emotion when another person has intentionally given, or attempted to give, something of value (Bartlett and DeSteno, 2006). Cicero regarded gratitude as the parent of all other virtues (Cicero, 1851, p. 139), and Roman stoic Seneca conceived of gratitude as a fundamental motivational drive critical for building interpersonal relationships (McCullough et al., 2001; Fox et al., 2015). In line with such assertions of early writers, several theorists have believed that gratitude nurtured social relationships through its encouragement of reciprocal, prosocial behavior between a benefactor and a recipient (Emmons and McCullough, 2003).

Reciprocal behavior is one of the research focuses of experimental and behavioral economics, and behavioral economists usually use the gift exchange model to analyze this behavior (Fehr et al., 1993, 1998). Fehr et al. (1993) constructed a labor market environment in laboratory and found that worker effort increased with wage. They interpreted this as evidence of fairness or reciprocity effects, given that workers could (anonymously and with impunity) have simply selected the minimum effort level after accepting a wage offer, which was what conventional economic theory predicted that self-interested and effort averse workers would do.

From the perspective of gratitude, Baron (2013) investigated the gratitude-based employment system by drawing upon recent works on reciprocity and gift exchange. He proposed the notion of “empathy wages,” in which the effect of the premium paid depended on the extent to which it elicited gratitude from recipients. He argued that prospects for eliciting gratitude were potentially greater in relative terms toward the bottom of the talent distribution, whereas creating equivalent feelings at the top was more difficult and costly. A field experiment among Canadian tree planters provided substantially informative data (Baron, 2013). Bellemare and Shearer (2009) showed that employees responded to gifts through discretionary effort, and this response appeared to have been markedly stronger among the least productive tree planters relative to other workers.

Psychological studies on gratitude have provided insights into its benefits. McCullough et al. (2001) proposed that gratitude was a moral affect with a moral motive function, which motivates a grateful person to behave prosocially toward a benefactor. Wood et al. (2008a) argued that gratitude was significantly related to the cognitive process of benefit appraisal. Despite recent findings on the effectiveness of gratitude intervention, the basic neural mechanisms involved in gratitude are relatively unknown (Kini et al., 2016). The investigation of the neural basis of gratitude would extend affective neuroscience beyond the study of basic emotions into complex social emotions essential for well-being.

The investigation of the experience and expression of gratitude is only the beginning at brain level (Fox et al., 2015). Zahn et al. (2014) determined that individual differences in gratitude tendencies correlated with gray matter volume in the right inferior temporal gyrus and posteromedial cortices. Algoe and Way (2014) found the correlation between the genotype for oxytocin function and the behavioral expressions of gratitude. Kini et al. (2016) examined the neural bases of gratitude expression and how gratitude expression might lead to long-term effects on brain activity. Their cross-sectional study indicated that a simple gratitude letter writing intervention improved lasting neural sensitivity to gratitude. Gratitude letter writing participants exhibited a high degree of behavioral gratitude and a significantly high neural modulation of gratitude in the medial prefrontal cortex (mPFC) 3 months later. Fox et al. (2015) conducted an experiment and induced gratitude in participants who underwent functional magnetic resonance imaging (fMRI). They suggested that gratitude ratings would correlate with activities in brain regions associated with moral cognition (mPFC and anterior cingulate cortex), reward (vmPFC) and theory of mind (dorsal mPFC).

Gratitude is a social emotion that signals our recognition of the things others have done for us (Emmons and McNamara, 2006). This emotion correlates with brain activity in circuits associated with social cognitive processes, such as perspective taking and theory of mind (Fox et al., 2015). Ortony et al. (1988) suggested that emotion was the product of cognitive systems. Social neuroscience findings showed that activity in the mPFC was linked to social cognitive, reward (Amodio and Frith, 2006), decision-making and evaluation processes (Tabibnia and Lieberman, 2007; Weber and Huettel, 2008), as well as emotion (Damasio et al., 1996). Thus, we hypothesized that gratitude would relate to changes in activity in the mPFC.

However, most studies on the neural correlates of gratitude have typically categorized individuals into homogeneous categories and have ignored the role of individual social heterogeneity (such as the ability of employees in the labor market). Results of behavioral studies, such as that of Baron (2013), have indicated that relatively disadvantaged and/or low performing individuals do appear more grateful (or inclined to reciprocate gifts) than high performers. Wood et al. (2008b) reported that cognitive and assessment processes were crucial to enable an individual to experience gratitude, and an individual with high trait gratitude would feel more state gratitude. Moreover, Markus et al. (1985) suggested that high masculinity was associated with bias in information processing that emphasizes the masculine characteristics of others, even when their behavior was irrelevant to the issue of masculinity. Individual social heterogeneity (e.g., status, endowment, ability and masculinity) and reference point played pivotal roles in individual value judgments (Kahneman and Tversky, 1979). Among such individual social heterogeneities, Baron (2013) showed that employee ability was closely correlated with gratitude. In the labor market, ability determined the status of employees in the employment relationship and the reference point of their expected salaries to some extent. High offers would more likely exceed the expectations of low-ability individuals and consequently induce feelings of gratitude, that is, individual social heterogeneity, particularly employee ability, vitally influences their experience of gratitude.

In this study, we constructed a labor market context and used the transcranial direct current stimulation (tDCS) technique to elucidate the correlation between mPFC and gratitude specifically and to explore the effect of the heterogeneous ability of employees; this ability is proven to be closely related to the gratitude of employees (Baron, 2013). We hypothesized that modulating mPFC activity will change the experience and expression of gratitude and that individual social heterogeneity (e.g., heterogeneous ability of employees in the labor market) will affect the feeling of gratitude and the correlation between mPFC and gratitude.

Most studies on gratitude have used stories or vignette methods (Fox et al., 2015; Simão and Seibt, 2015), where participants were asked to place themselves in a specific context and imagine what they would feel. However, text-based approach might have a ceiling or floor effect problem while triggering emotional responses (Tsang, 2007). Thus, the accuracy of this approach is susceptible to social praise effects (Pedregon et al., 2012). Other scholars have utilized gratitude letter writing or keeping a gratitude diary to analyze gratitude (Algoe et al., 2010), which are difficult to quantify. Moreover, Algoe et al. (2008) examined the role of gratitude in actual ongoing relationships. In addition to the previously presented methods, several studies have induced gratitude toward a stranger through a laboratory experiment to obtain a high degree of experimental control (Leung, 2012). Behavioral game experiment places participants in a specific interactive environment with monetary incentive, wherein they behave according to their will. We believe that the gratitude of participants could be well investigated with a reasonable experimental design.

Thus, we designed a variant of gift exchange game, based on the studies of Fehr et al. (1993) and Baron (2013), to verify our hypotheses. Before the experiment, we divided participants into high- and low-ability groups using a task with certain difficulty. Participants were asked to join the experiment by playing the role of employee. Empirical literature on gratitude provided substantial findings on whether grateful individuals would repay a benefactor or a fortunate bystander (Bartlett and DeSteno, 2006). The link was relatively strong that repayment behavior was sometimes considered to imply feelings of gratitude (Algoe et al., 2008). Therefore, in our study, we used the efforts of participants to represent their levels of gratitude. Neuroimaging studies of Fox et al. (2015) and Kini et al. (2016) suggested that the mPFC is an important brain region for experiencing and expressing gratitude. Prior to the experiment, we used the tDCS technique to stimulate the mPFC. Each participant randomly received one of anodal, cathodal, or sham stimulation. This approach allows us to measure the different effects of modulating the mPFC on low- and high-ability participants. We obtained two main results: First, the gratitude levels of low-ability employees were significantly higher than those of high-ability employees in the sham stimulation group. Second, compared with the sham stimulation, the anodal tDCS of the mPFC significantly increased the gratitude levels of high-ability employees, whereas the cathodal tDCS of the mPFC decreased the gratitude levels of low-ability employees. We investigated the relationship between mPFC and gratitude using the tDCS technique and provided neural evidence for the incentive mechanism design in the labor market.

## Materials and Methods

### Participants

A total of 89 healthy young people (mean age of 20.3 years old, ranging from 17 to 24; 49 females, 40 males) were recruited from the undergraduate and graduate student population of Nankai University. Participants were right-handed with normal or corrected normal vision. They have no previous knowledge of the tDCS technique or experience in a gift exchange game experiment. None of the participants reported a history of neurological or psychiatric problems. The experiment lasted approximately 60 min. Each participant received a payment of approximately 65 Chinese Yuan (approximately 10 US dollars). Participants did not report any adverse side effects, such as scalp pain or headaches, after the experiment. All of them provided written informed consent, and the research was approved by the Ethics Committee of Nankai University. The experiment was performed in accordance with the Declaration of Helsinki and was approved by the Ethics Committee of Business School of Nankai university.

### tDCS

In the tDCS technique, a direct current of low-level intensity (1–2 mA) is applied for a few minutes via electrodes placed on the subject’s scalp. This current reaches the cortex and modulates the membrane polarity of neurons within a region of underlying neural tissue. tDCS-induced changes during stimulation are caused by changes in the permeability of the neural membrane, which is depolarized by anodal stimulation and hyperpolarized by cathodal stimulation (Nitsche and Paulus, 2000; Stagg and Nitsche, 2011). Therefore, tDCS can transiently influence behavior by altering neuronal activity, which may have facilitatory or inhibitory behavioral effects.

**Figure 1:**
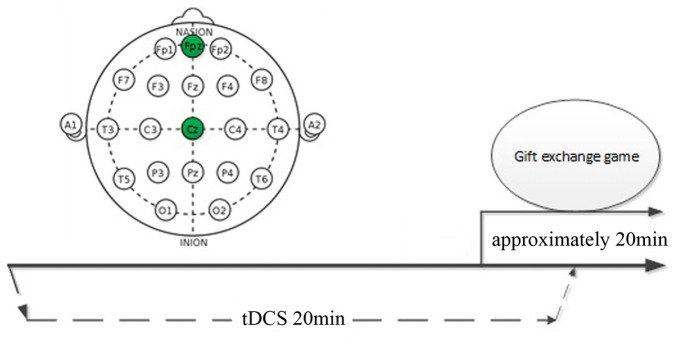
Schematic representation of the experimental design.

Transcranial direct current is generated by a battery-driven constant current stimulator (DC-Stimulator, NeuroConn, Germany), whereas anodal and cathodal stimulation electrodes are inserted into a 5 cm^2^ × 7 cm^2^ physiological saline-soaked sponge. In our study, participants were blinded to the stimulation (single-blinded design) and randomly assigned to one of three groups, namely, mPFC anodal (*n* = 29; 16 females), mPFC cathodal (*n* = 27; 15 females), and sham (*n* = 33; 18 females). None of the participants reported any previous knowledge of the tDCS technique and any experience in stimulation. Moreover, none of the participants were aware of the type of stimulation they received, whereas the experimenter was fully informed. According to the International 10–20 EEG System, in the anodal stimulation group, the anode was placed at Fpz (Civai et al., 2015) and the cathode was placed at Cz (see Figure 1). In the cathodal stimulation group, the cathode was placed at Fpz and the anode at Cz. The sham stimulation group was similar to the anodal stimulation group, except for the stimulation current that lasted only 30 s. Participants may experience the initial micro itch, but differentiating sham stimuli from real stimuli is difficult (Gandiga et al., 2006). According to Civai et al. (2015), the two stimulus currents were fixed at 2 mA for 20 min with a 15 s rise and fall time. Previous studies have shown that the intensity of 0.057 mA/cm^2^ and total charge of approximately 0.0063 C/cm^2^ were safe and well tolerated (Minhas et al., 2010; Borckardt et al., 2012). Moreover, specifying that the tDCS was not focal was important; thus, the simulation effects were more widespread and unclearly confined to the area identified by an imaging study; however, the area under the electrode could be assumed to be most affected by the stimulation (Civai et al., 2015).

### Task and Procedure

The experiment is a revised gift exchange game. Classical gift exchange game has two roles, namely, employer and employee. One employer and one employee constitute a group and interact with each other through the entire experiment. First, the employer has a certain amount of initial endowment *g* and decides to give a certain amount of wage *w* to the employee. The employee then selects the degree of effort *e*, which generates a certain cost *c*(*e*) to him/her. The employer obtains an income of *g* − 100*e* from the employee’s effort, whereas the employee earns *w* − *c*(*e*).

In our experiment, all participants played the role of employees. Employers acted through computers, which was unknown to the participants. Baron (2013) suggested that the following factors would affect the gratitude of employees: (1) ability level: low-ability employees were tended to be grateful; (2) comparison between expected and real wage: unexpected wage could make employees more grateful; and (3) initial reference point: high wages would induce the gratitude of employees when their initial wages were low. Therefore, we classified participants into two types (i.e., low and high ability) by asking them to finish a task, i.e., answer nine questions (selected from the civil service exam test question bank, including three semantic, mathematical, and inference questions each) in 18 min before the experiment. Each question was worth 10 points. When the results were obtained, the participants were asked to answer two other questions, as follows: What do you think about your performance? How much would you like the employer to pay you? According to the results, employees whose scores were between 0 and 30 were classified as low ability, whereas employees with scores of 40 and 60 were categorized as high ability. No participants’ scores were more than 70. The expected wages of the two types of employees were significantly different.

In our experiment, employers (computers) received 100 G$ at the start of each trial. Employees received no initial endowment. The test score of the employee was displayed on the screen. The employer selected wage *w* (an integer from 0 to 100, an arithmetic sequence with the interval of 10) after seeing the score of the employee. When the employee saw the wage given by the employer, he/she selected the degree of effort *e* (a decimal number between 0 and 1), and the effort would generate a certain cost *c*(*e*) to him/her (see Table 1). The employer could see the effort of the employee and obtain an income of *xe*, with *x* determined by the score of the employee ([0, 30], *x* = 30; [40, 60], *x* = 60; [70, 90], *x* = 90). The final incomes of the employer and employee were 100 − *w* + *xe* and *w* − *c*(*e*), respectively. The wages given by the employers (computers) were gradually increased (see Table 2). The pretest showed that this setup could successfully induce the gratitude of participants and that repeated wages could verify the stability of the participants’ behavior.

**Table 1:** Cost of the effort of employees.

*E*	0.1	0.2	0.3	0.4	0.5	0.6	0.7	0.8	0.9	1
*c*(*e*)	0	1	2	4	6	8	11	14	17	20

**Table 2:** Wages in 15 periods.

Period	1	2	3	4	5	6	7	8	9	10	11	12	13	14	15
W	10	10	20	20	30	30	40	40	50	50	70	60	50	60	50

*Note: wages were exogenous and the same for all employees for comparison*.

## Results

### Behavioral Data

Behavioral data were statistically evaluated using the SPSS software (version 22; SPSS Inc., Chicago, IL, USA). The significance level was set at 0.05 for all analyses. We considered the effort levels of employees to represent degrees of gratitude. We analyzed the mean effort levels of participants with different abilities between three stimulation groups. A *post hoc* one-way analysis of variance (ANOVA) at stimulation type exhibited significant difference across any stimulation type (*F* = 3.45, *p* = 0.036). In the case of significant effects, *post hoc* Student’s paired *t* tests were conducted to examine whether an active intensity resulted in a significant difference relative to the sham stimulation in subsequent analyses.

### General tDCS Effect on the mPFC of Employees’ Gratitude

**Figure 2:**
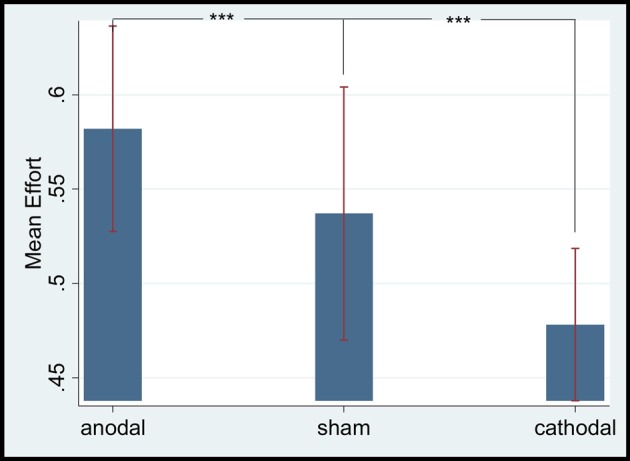
Mean effort levels of different stimulation groups. ***Means significant difference at 1% level (*P* < 0.01).

According to Baron (2013), employees would feel gratitude when the wage paid by employers exceeded their expectation. The current data showed that the mean expected wage of low-ability participants was 38.83 (SD = 16.73, max = 70, min = 10), whereas that of high-ability participants was 53.89 (SD = 15.10, max = 80, min = 30). Thus, we only analyzed scores greater than 30 (i.e., from periods 7–15) in the subsequent part. The mean effort levels of the anodal, sham, and cathodal stimulation groups were 0.581 (SD = 0.25, max = 1, min = 0.2), 0.537 (SD = 0.24, max = 1, min = 0.1) and 0.478 (SD = 0.27, max = 1, min = 0.1), respectively. The mean effort level of the anodal stimulation group was significantly higher than that of the sham (*t* = 4.28, *p* = 0.0013, paired *t* test) and cathodal stimulation groups (*t* = 8.2466, *p* = 0.0000, paired *t* test). The mean effort level of the cathodal stimulation group was significantly lower than that of the sham stimulation group (*t* = −3.164, *p* = 0.0067, paired *t* test; see Figure 2).

### Effect of tDCS on the mPFC of Homogeneous Employees’ Gratitude

**Figure 3:**
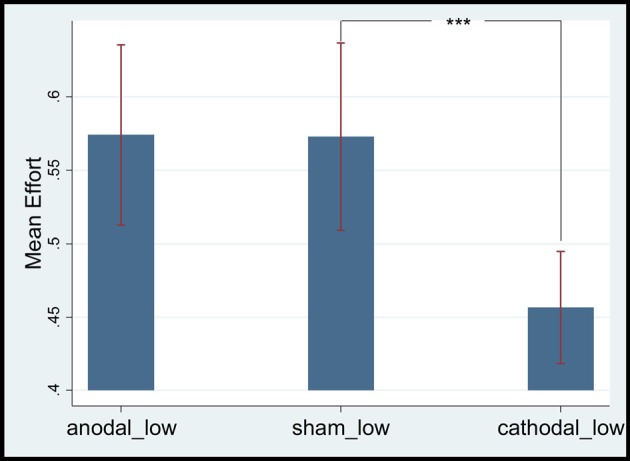
Mean effort levels of low ability employees. ***Means significant difference at 1% level (*P* < 0.01).

The study included 44 low- (15 anodal, 12 cathodal, and 17 sham) and 45 high-ability participants (14 anodal, 15 cathodal and 16 sham). We compared the mean effort levels of participants with different abilities. First, we analyzed the effect of mPFC stimulation on the gratitude of low-ability employees. Their mean efforts in the anodal, sham, and cathodal groups were 0.574 (SD = 0.28, max = 1, min = 0.1), 0.572 (SD = 0.20, max = 1, min = 0.1) and 0.456 (SD = 0.23, max = 0.8, min = 0.1), respectively. The mean effort level of the anodal stimulation group was higher than that of the cathodal stimulation group (*t* = 5.5156, *p* = 0.0003, paired *t* test), whereas the mean effort level of the cathodal stimulation group was significantly lower than that of the sham stimulation group (*t* = 6.2499, *p* = 0.0001, paired *t* test). No significant difference in effort levels between anodal and sham stimulation groups was observed (*t* = 0.1105, *p* = 0.4574, paired *t* test; see Figure 3).

**Figure 4:**
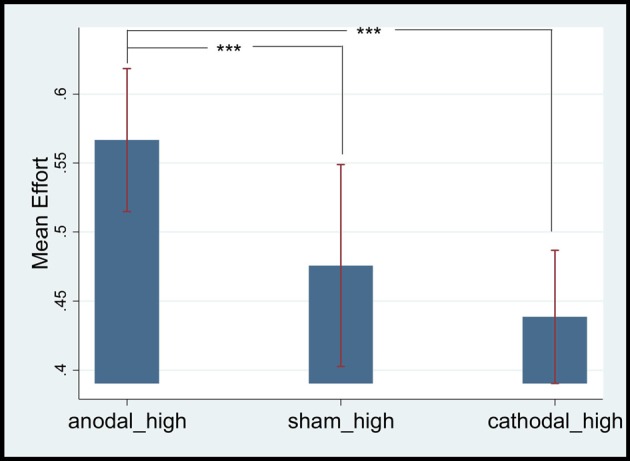
Mean effort levels of high ability employees. ***Means significant difference at 1% level (*P* < 0.01).

Second, the effect of mPFC stimulation on the gratitude of high-ability employees was analyzed. Their mean efforts in the anodal, sham, and cathodal groups were 0.567 (SD = 0.25, max = 1, min = 0.1), 0.47 (SD = 0.26 max = 1 min = 0.1), and 0.45 (SD = 0.24, max = 1, min = 0.1), respectively. The mean effort level of the anodal stimulation group was significantly higher than that of the cathodal (*t* = 7.2737, *p* = 0.0000, paired *t* test) and sham stimulation groups (*t* = 4.3040, *p* = 0.0013, paired *t* test). No significant difference in effort levels between cathodal and sham stimulation groups was observed (*t* = 1.7264, *p* = 0.0613, paired *t* test; see Figure 4).

### tDCS Effect on the mPFC of the Gratitude of Employees with Heterogeneous Ability

**Figure 5:**
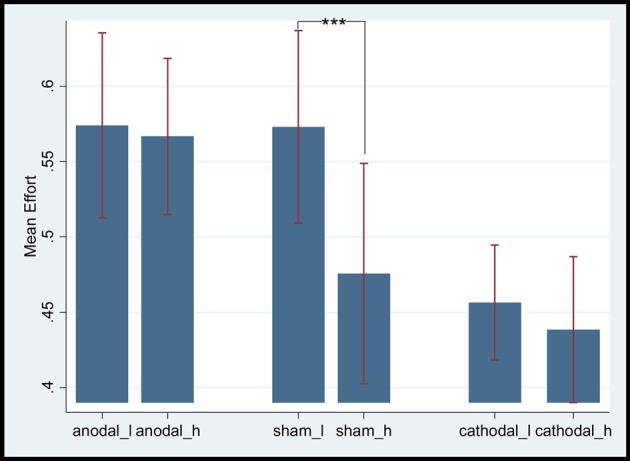
Mean effort levels of two type employees in three simulation group. ***Means significant difference at 1% level (*P* < 0.01).

We compared the effort levels between low- and high-ability employees under the same stimulation. No significant difference in the effort level between low-and high-ability employees was observed in the cathodal (*t* = 1.0934, *p* = 0.1530, paired *t* test) and anodal stimulation groups (*t* = 0.4456, *p* = 0.3338, paired *t* test). In the sham stimulation group, the mean effort level of low-ability employees was significantly higher than that of high-ability employees (*t* = 6.9353, *p* = 0.0001, paired *t* test; see Figure 5).

Combing the results above, we suggested: (1) the gratitude degree of low-ability employees was significantly higher than that of high-ability employees; (2) the anodal stimulation of mPFC significantly improved the gratitude degree of high-ability employees, but indicated no significant effect on the gratitude of low-ability employees; and (3) the cathodal stimulation of mPFC decreased the gratitude degree of low-ability employees, but failed to significantly affect the gratitude of high-ability employees (see Table 3).

**Table 3:** Summary of the results.

Ability type	All	Low	High
Gratitude level	Anodal ≻ sham ≻ cathodal	Anodal ≈ sham ≻ cathodal	Anodal ≻ sham ≈ cathodal
Stimulation	Anodal	Sham	Cathodal
Gratitude level	Low ≈ high	Low ≻ high	Low ≈ high

*Note: this table summarizes the effects of mPFC stimulation on gratitude; “≻” represents “higher than,” “≈” represents “no significant difference between”*.

## Discussion

Previous fMRI studies (Fox et al., 2015; Kini et al., 2016) have shown that an increase in mPFC activation was specifically associated with the experience and expression of gratitude. In this work, we obtained converging evidence using a complementary technique (i.e., tDCS), in which we modulated the gratitude level of employees through a stimulus applied to the mPFC for 20 min.

On the effects of tDCS, although the concept of tDCS changing performance seemed well established for tDCS in the motor system (Stagg and Nitsche, 2011), the same concept was not so directly applicable in the cognitive neuroscience field; furthermore, the relationship between type of stimulation and final behavior was often quite complex (e.g., Jacobson et al., 2012; Miniussi et al., 2013). The current data showed that anodal (cathodal) stimulation of the mPFC increased (decreased) the effort level of employees compared with sham stimulation, which might imply that anodal (cathodal) stimulation could facilitate (inhibit) the excitability of mPFC. In our study, the stimulation current was fixed at 2.0 mA, based on the study of Civai et al. (2015). Batsikadze et al. (2013) proposed that enhanced tDCS current intensity did not necessarily increase the efficacy of cathodal stimulation, but might shift the direction of excitability alterations. However, Jamil et al. (2017) showed that the effect of 2.0 mA cathodal stimulation for 20 min differed with sham stimulation, although not significant and did not shift the excitability direction. They proposed that intensities of approximately 1.0 mA might be optimal in inducing the strongest inhibition of motor cortical excitability in healthy adults. In a study of adolescents, 10 min of 0.5 mA cathodal tDCS (35 cm^2^ electrodes) significantly decreased cortical excitability, but 1.0 mA cathodal tDCS increased cortical excitability (Moliadze et al., 2015). Civai et al. (2015) used 2.0 mA cathodal stimulation over mPFC and found that cathodal stimulation decreases the probability of rejecting unfair offers compared with the baseline. Shen et al. (2016) applied 2.0 mA cathodal stimulation to the left dlPFC using HD-tDCS and obtained a significant treatment effect. No consistent conclusion about the effect of tDCS intensity has yet been achieved. Thus, the only way to accurately determine what happens to mPFC functionality under a certain stimulation involves the collection of imaging data during, or soon after, stimulation. Further studies should combine these techniques to obtain detailed answers. The low spatial focality of tDCS due to heterogeneous tissue conductivities should also be considered (Nitsche and Paulus, 2011). We believe that the effects of the tDCS in this study should be interpreted in terms of effects on mPFC, which was the area under the electrode.

Emotional cognitive theory accounts for the involvement of mPFC in gratitude, indicating emotion as the product of cognitive systems (Ortony et al., 1988). An increasing number of neuroimaging studies have proposed emotional evaluation as one of the important functions of mPFC (Knutson et al., 2001; McClure et al., 2004; Harris et al., 2007). Arnold (1968) argued that the nature of emotions was indirectly determined by stimulus scenarios and that subjective evaluation played a mediating role between stimuli and emotions. The basic process was as follows: stimulating scenarios → assessment → emotions. Gratitude is a social emotion that signals our recognition on what others have done for us (Emmons and McNamara, 2006). In the cognitive process of gratitude, recipients evaluate the value of benevolence and the motivation of the benefactors (Algoe, 2012). Such evaluation is also affected by the characteristics of the beneficiaries and the relationship between benefactors and beneficiaries. Thus, different assessments of the same stimulus scenario would result in different emotional responses. Harris et al. (2007) suggests that affective evaluation may be a general function of mPFC. Using the tDCS technique to modulate mPFC activity would affect cognition and evaluation processes, ultimately influencing the feeling of gratitude, which could explain the conclusion of our study to a certain extent.

In this work, we verified the role of individual social heterogeneity (e.g., ability in the labor market) in the experience of gratitude and the correlation between mPFC and gratitude, which has been ignored by most neurological studies on emotion. The results of one-way ANOVA on behavioral data indicated no significant difference in the effort level between low- and high-ability employees. However, subsequent analysis showed that a significant difference in the effort level between low-ability and high-ability employees was observed in the sham stimulation group, which was consistent with the findings of Baron (2013). The cathodal stimulation of mPFC significantly reduced the gratitude level of low-ability employees, but has no significant effect on high-ability employees. By contrast, the anodal stimulation of the mPFC significantly increased the gratitude level of high-ability employees, but has no evident effect on low-ability employees. These findings indicated that the effect of modulating mPFC activity on gratitude might be different for heterogeneous individuals. Thus, different effects of tDCS on the mPFC of different employee types diminished the difference of gratitude between them.

Psychological research on counterfactual reasoning has accounted for the expected disproportionate response from less advantaged workers (Medvec et al., 1995; Medvec and Savitsky, 1997). Related literature has demonstrated how gratitude or satisfaction reflected not only one’s absolute outcomes but also how those outcomes compared with what the person might have otherwise plausibly expected (Baron, 2013). In our experiment, participants were asked to recognize the classification and perform cognition about the relationship between ability and wage. We then let them write down their expected wages. Results showed that the mean expected wage of high-ability employees was significantly higher than that of low-ability employees (mean *H* = 53.89, mean *L* = 38.83, *t* = 4.20, *p* = 0.000, independent sample *t* test). Differences in expected wage would affect feeling of gratitude. Findings indicated that low-ability employees would be more sensitive to feeling gratitude. Tesser et al. (1968) found that the higher the assessment of the cost of assistance and the value of favor, which were determined by the endowment of beneficiaries, the higher the gratitude degree that the employees felt. Baron (2013) suggested that ability would influence the gratitude of employees and affect their behavior in turn. Results showed that unexpected wages would make employees more grateful and motivate them to exert stronger efforts, whereas high wages could not induce the gratitude of high-ability employees successfully and the effect of incentive was obscure. Our behavioral results confirmed the findings of Baron (2013). The findings on the stimulation effects on employees with heterogeneous ability provided neural evidence for the incentive mechanism design in the labor market.

We obtained interesting and unexpected findings. No difference in effort levels of low-ability participants between anodal and sham stimulations and of high-ability participants between cathodal and sham stimulation were observed. Jacobson et al. (2012) assumed that the direct current might have different effects depending on the neuronal state and initial activation level in the stimulated regions. This finding may be attributed to the activation level in the mPFC of low-ability (high-ability) participants which might be sufficiently high (low) when facing the stimulus scenarios in our experiment. Stimulation effects might be affected by the ceiling or floor effect. However, exploring more detailed answers by combining fMRI and tDCS techniques in further studies is necessary.

Our study included several limitations. Given the use of a multistage repeated experimental framework, reputation and signal transmission might have affected the behavior of participants. de Quervain et al. (2004) argued that gratitude expression could be used to convey reciprocal promises to prevent social punishment as a free rider and to signal that they were fair partners to others (Sigmund, 2007). The significant treatment effects of stimulation under the same experimental design would keep the results robust. Further studies should consider the potential effect of these factors. Meanwhile, exploring the neural mechanisms of emotion- and strategy-driven behaviors would be interesting.

## Conclusion

In summary, our findings provided important information about the effect of tDCS on healthy participants, particularly with respect to the gratitude degrees of individuals with different abilities. Activating mPFC by tDCS could affect the gratitude degree in a gift exchange game. The gratitude degree of participants under anodal stimulation was higher than that under cathodal stimulation. Moreover, anodal stimulation could increase the gratitude degree of high-ability participants, whereas cathodal stimulation could decrease the gratitude degree of low-ability participants.

## Author Contributions

PW, JL, GW and XN designed experiment; PW, JL and XN performed experiment; PW and HS analyzed data and wrote the manuscript.

## Conflict of Interest Statement

The authors declare that the research was conducted in the absence of any commercial or financial relationships that could be construed as a potential conflict of interest.
